# Elevated ApoC3 levels in cerebrospinal fluid predict poor outcomes in patients with aneurysmal subarachnoid hemorrhage

**DOI:** 10.3389/ebm.2026.10827

**Published:** 2026-04-17

**Authors:** Bin Tong, Junjie Wang, Jiarui Chen, Qia Zhang, Zhouhan Xu, Kaichuang Yang, Xiaomin Chen

**Affiliations:** 1 Department of the Operating Room, Zhejiang Provincial People’s Hospital, Affiliated People’s Hospital, Hangzhou Medical College, Hangzhou, China; 2 Department of Neurosurgery, Second Affiliated Hospital, School of Medicine, Zhejiang University, Hangzhou, China; 3 Center for Rehabilitation Medicine, Department of Neurosurgery, Zhejiang Provincial People’s Hospital, Affiliated People’s Hospital, Hangzhou Medical College, Hangzhou, China; 4 Department of Nursing, Zhejiang Provincial People’s Hospital, Affiliated People’s Hospital, Hangzhou Medical College, Hangzhou, China

**Keywords:** aneurysmal subarachnoid hemorrhage, ApoC3, cerebrospinal fluid, delayed cerebral ischemia, hydrocephalus

## Abstract

Aneurysmal subarachnoid hemorrhage (aSAH) is a devastating condition associated with approximately 30% mortality and 20% severe disability among survivors. Delayed cerebral ischemia due to cerebral vasospasm and hydrocephalus significantly contribute to poor neurological outcomes. Currently, reliable biomarkers for early prediction of these complications remain lacking. In this study, 63 patients with a mean age of 59.7 ± 11.53 years were enrolled. Functional outcomes were assessed by the modified Rankin Scale (mRS). Cerebrospinal fluid (CSF) samples were obtained through lumbar drainage (LD) or external ventricular drainage (EVD) and analyzed by ELISA. The predictive value of biomarkers was evaluated using receiver operating characteristic (ROC) curve analysis. Elevated Apolipoprotein C-III (ApoC3) levels in CSF of aSAH patients were observed. Furthermore, increased ApoC3 concentrations were significantly associated with poor prognosis and an elevated risk of severe complications. At an optimal cutoff value of 4,463 ng/mL, patients with high ApoC3 levels exhibited significantly worse 3-month functional outcomes and a higher incidence of delayed cerebral ischemia and hydrocephalus. Monitoring ApoC3 levels in CSF may be beneficial for predicting complications such as delayed cerebral ischemia and hydrocephalus in patients with aSAH.

## Impact statement

Although surgical clipping or endovascular coiling, alongside vasospasm management have improved survival rates, the rates of morbidity and mortality of Subarachnoid hemorrhage (SAH) patients remain unacceptably high. Early diagnosis and timely intervention are paramount for optimizing outcomes. In our study, we found that CSF ApoC3 levels in aSAH patients were significantly negative correlated with clinical scores. This study may improve prognostic assessments, enable early identification of high-risk patients, and potentially guide personalized treatment strategies, ultimately reducing the morbidity and mortality associated with SAH and uncovering new therapeutic targets for managing this condition.

## Introduction

SAH is a severe form of stroke characterized by bleeding into the subarachnoid space, usually caused by the rupture of an intracranial aneurysm. Although SAH represents only 5%–10% of all stroke cases, it is associated with disproportionately high mortality and long-term disability rates [1]. Globally, the incidence of SAH ranges from 6 to 10 cases per 100,000 people annually, typically occurring between ages 50 and 60, with a slight predominance in females [1]. Despite advances in medical treatment, the case-fatality rate remains alarmingly high, with 40%–50% of patients succumbing within the first month following SAH [2]. Even with current therapeutic strategies, survivors have significantly different prognoses. Approximately 10%–20% of patients experience severe disabilities, including lasting cognitive and physical impairments that limit independence and quality of life. In contrast, about 63.3% of patients without infarction achieve excellent outcomes (mRS scores of 0–1) within 1 year [2, 3]. Currently, clinical management strategies are uniform across SAH patients. Given the substantial variability in outcomes and the clear relationship between excellent outcomes and the absence of infarction, reliable prognostic biomarkers are essential. Such biomarkers could identify patients at high risk for poor outcomes and facilitate early interventions to manage brain edema and prevent vasospasm, thereby altering disease progression and improving prognosis. However, reliable biomarkers are currently lacking.

The pathophysiology of SAH is complex, involving primary and secondary mechanisms of injury. The initial hemorrhagic event initiates multiple complications, including cerebral vasospasm, brain edema, intracranial hypertension, and a robust inflammatory response due to blood accumulation in the subarachnoid space. Vasospasm occurs in up to 70% of patients, resulting from endothelial dysfunction, and leads to delayed cerebral ischemia in 30%–40% of cases, significantly contributing to poor neurological outcomes [4, 5]. Additionally, brain edema and elevated intracranial pressure further exacerbate secondary injuries, complicating clinical management [5].

ApoC3 is a glycoprotein primarily involved in lipid metabolism, particularly the regulation of triglyceride-rich lipoproteins (TRLs) such as very low-density lipoproteins (VLDL) and chylomicrons [6]. ApoC3 slows the catabolism of these lipoproteins by inhibiting lipoprotein lipase (LPL) activity, contributing to hypertriglyceridemia [6]. Beyond its metabolic functions, ApoC3 is implicated in inflammation and endothelial dysfunction, crucial mechanisms in cardiovascular and cerebrovascular diseases [6, 7].

As mentioned previously, vasospasm significantly contributes to poor neurological outcomes [8]; however, no reliable biomarkers currently exist to predict vasospasm in SAH patients. Considering ApoC3’s established role in inflammation and endothelial dysfunction, ApoC3 may serve as a promising prognostic biomarker.

Given its known involvement in cerebrovascular diseases, we hypothesize that ApoC3 levels represent effective biomarkers for predicting vasospasm and overall prognosis in SAH patients. Specifically, ApoC3 may influence recovery by regulating inflammation, oxidative stress, and endothelial repair, processes essential for vascular healing and neurological improvement. To test this hypothesis, we aimed to evaluate the correlation between CSF ApoC3 levels and clinical outcomes in SAH patients, including the mRS score, cerebral vasospasm, and hydrocephalus. Since SAH frequently requires invasive procedures such as CSF analysis for diagnosis and monitoring, identifying CSF ApoC3 levels as a prognostic biomarker could offer valuable insights into disease progression and potentially reduce repeated invasive procedures. Results demonstrated that ApoC3 levels were an independent biomarker for SAH prognosis and significantly correlated with vasospasm and hydrocephalus. Early identification of SAH patients with elevated CSF ApoC3 levels and targeted interventions, such as enhanced vasospasm prevention, close monitoring of hydrocephalus through CT imaging, and prompt treatment, may help alter disease progression and improve patient prognosis.

## Materials and methods

### Study overview

This prospective observational cohort study enrolled patients with spontaneous aneurysmal SAH admitted to the Second Affiliated Hospital of Zhejiang University and Zhejiang Provincial People’s Hospital between October 2021 and October 2023 (ClinicalTrials.gov NCT06009016). Eligible patients were required to be ≥18 years old, diagnosed with aneurysmal SAH, and have a modified Fisher score (mFS) of 2-4 by Computed Tomography Angiography (CTA) within 24 h of symptom onset. Exclusion criteria included: (1) non-aneurysmal SAH (e.g., trauma, arteriovenous malformations, angiogram-negative SAH); (2) previous central nervous system (CNS) disorders (stroke, traumatic brain injury, CNS infections); (3) severe comorbidities within 6 months prior to SAH (e.g., malignancies, drug-resistant cardiovascular diseases, coagulation disorders, or organ dysfunction). Patients with normal pressure hydrocephalus (NPH) aged ≥18 years were enrolled as the control group, presenting at least two symptoms of the Hakim-Adams triad (gait disturbance, urinary incontinence, and cognitive decline), CSF pressure <200 mm H_2_O, normal CSF content, and exclusion of other medical causes [9]. Ethical approval was granted by the Institutional Review Board of the Second Affiliated Hospital of Zhejiang University (Approval No. 2023-059). Informed consent was obtained from participants or their family members, or waived by the board.

### Treatment protocol

Digital subtraction angiography (DSA) was performed within 24 h of admission to identify the responsible aneurysm, followed by embolization or clipping by experienced neurointerventional or neurosurgical teams. LD was routinely performed for patients with an mFS of 3-4, except when acute hydrocephalus necessitated EVD. Postoperative brain CT scans were initially performed, with follow-up scans every 1–3 days, as clinically indicated, until discharge. Nimodipine was administered to prevent cerebral vasospasm, and intravenous rehydration was used to maintain normal blood volume.

### Sample size calculation

Sample size estimation was conducted by G*Power (version 3.1) to determine the number of patients of SAH (one-sample case), with an α error probability of 0.05, effect size [d] of 0.4, study power (1-β) of 0.9. The result was 55 aSAH patients. We totally enrolled 63 patients to ensure the study’s statistical power.

### mRS outcome assessment

Functional outcomes assessed by the mRS were collected via telephone calls or hospital and clinic visits 3 months after discharge. mRS scores of 0–2 were considered good outcomes, while scores of 3–6 indicated poor outcomes. Assessments were conducted by independent, trained investigators blinded to study details to minimize bias.

### Data collection

Baseline characteristics (age, gender, BMI, medical history, and social history) were assessed by the treating physician. Clinical data upon admission, including the Glasgow Coma Scale (GCS), Hunt and Hess (HH) grade, and World Federation of Neurosurgeons Scale (WFNS), were recorded. Treatment-related data, such as surgical approaches, ICU length of stay, and duration of mechanical ventilation, were also documented. SAH-related complications, including seizures and delayed cerebral ischemia (DCI), were monitored. DCI was defined as focal neurological deficits or a ≥2-point decline in GCS, caused by cerebral vasospasm or infarction (new infarcts on CT or MRI, excluding those occurring within 48 h post-surgery). Functional outcomes were evaluated at 3 months post-discharge using the mRS, with good prognosis defined as mRS scores of 0–3 and poor prognosis as scores of 4–6. Radiological data collected from head CT scans included mFS, Subarachnoid Hemorrhage Early Brain Edema Score (SEBES), intraventricular hemorrhage (IVH), and the highest Hounsfield unit (HU) in the hemorrhagic area. CSF samples were collected via LD or EVD within 72 h postoperatively. Following collection, samples were centrifuged at 3,000 rpm for 10 min at 4 °C to remove erythrocytes and immune cells, and stored at −80 °C until ELISA analysis.

### ELISA detection

CSF ApoC3 concentrations were determined using the Human Apolipoprotein CIII ELISA Kit (ab154131, Abcam, Cambridge, MA, USA) according to the manufacturer’s protocol. The intra-assay coefficient of variation (CV) is 6% and the inter-assay CV is 11%. All samples were tested in duplicate, and mean values were calculated for further analysis. Samples were analyzed on two plates within the same day to minimize variability between assays. A standard curve was generated using a four-parameter logistic regression model, and ApoC3 concentrations were calculated by interpolation. All experiments and analyses were conducted by separate investigators, blinded to patients’ clinical information and 3-month outcome status. Sample identifiers were anonymized prior to analysis.

### Statistical analysis

Descriptive statistics are presented as counts (n) and percentages (%) for categorical variables, and mean ± standard deviation (SD) for continuous variables. Differences in patient characteristics were evaluated using unpaired Student’s t-tests or non-parametric Mann-Whitney U tests for continuous variables, and Pearson chi-square or Fisher’s exact tests for categorical variables. Normality of the data was assessed using the Shapiro-Wilk test. The predictive value of ApoC3 for mRS outcomes was assessed by ROC curve analysis, with optimal cutoff points determined by the Youden index. Statistical analyses were conducted using GraphPad Prism 8.2.1 (GraphPad Software, San Diego, CA, USA) and SPSS 23.0 (IBM, Armonk, NY, USA). A p-value <0.05 was considered statistically significant.

## Results

### Study population

A total of 63 patients with aSAH were enrolled between June 2021 and March 2023. Of these, 54% were female, with a mean age of 59.7 ± 11.53 years. The median hospital length of stay (LOS) was 12 days (IQR: 10–22 days), and the median neurosurgical intensive care unit (NSICU) stay was 6 days (IQR: 2–18 days). At admission, 61.9% of patients had a Glasgow Coma Scale (GCS) score of 13–15, whereas 20.6% had scores of 3–5. Regarding severity scales, 46% of patients had WFNS grades 3–5, and 44.4% presented with Hunt & Hess grades 3-4. Smoking and hypertension were common comorbidities, observed in 33.3% and 57.1% of patients, respectively. CSF examination showed a median nucleated cell count of 442 × 10^6^/L (IQR: 169–1,487) and a median protein level of 128.3 mg/dL (IQR: 69–157.6). The median CSF ApoC3 concentration was 3,149.5 ng/mL (IQR: 1,174.77–7,768.51). Aneurysms most frequently involved the anterior cerebral artery (49.2%), with sizes predominantly between 5.1 and 10 mm (52.4%). Regarding treatment, 66.7% underwent aneurysm clipping, and 33.3% received endovascular coiling. Post-treatment complications included clinical vasospasm in 36.5% and delayed infarction in 42.9% of patients. At 3-month follow-up, 47.6% of patients had poor outcomes (mRS scores 3–6). Detailed demographic and clinical data are presented in Table 1. The demographic characteristics of NPH patients are shown in Supplementary Table 1.

**TABLE 1:** Demographic and Clinical Data of 63 patients with aSAH.

Variable		Average/median/N	SD/IQR/%
Hospital LOS, d		12	(10–22)
Sex (F)		34	54%
Age, y		59.7	±11.53
Systolic blood pressure, mmHg		150	(137–168)
Systolic blood pressure >190		11	17.5%
Admission GCS	13–15	39	61.9%
	6–12	11	17.5%
	3–5	13	20.6%
WFNS grade (3-5)		29	46%
Hunt & hess grade (3-4)		28	44.4%
NSICU LOS, d		6	(2–18)
Duration of mechanical ventilation, d		5	(2–12)
Drinking		20	31.7%
Smoking		21	33.3%
Hypertension		36	57.1%
Diabetes		4	6.3%
BMI	<18.5	4	6.3%
	<24	12	50.8%
	<28	24	38.1%
	≥28	3	4.8%
CSF examination	ApoC3, ng/mL	3,149.5	(1,174.77–7,768.51)
	RBC, 10^6/L	83,000	(29,000–102000)
	Lymphocytes, %	29.26	(13–38)
	Nucleated cells, 10^6/L	442	(169–1,487)
	Glucose, mmol/L	3.82	(2.93–4.25)
	Protein level, mg/dL	128.3	(69–157.6)
	Cl^−^, mmol/L	129.5	(125.2–131.4)
mFS (3-4)		41	65.1%
SEBES (3-4)		46	73%
IVH		44	69.8%
mGS		2	(0–9)
Aneurysm Location	Internal carotid artery	5	7.9%
	Anterior cerebral artery	31	49.2%
	Middle cerebral artery	11	17.5%
	Posterior circulation	16	25.4%
Aneurysm size	1–5 mm	24	38.1%
	5.1–10 mm	33	52.4%
	Greater than 10 mm	6	9.5%
Coiling		21	33.3%
Clipping		42	66.7%
Decompression		11	17.5%
LD		43	68.3%
EVD		15	23.8%
Clinical vasospasm		23	36.5%
Delayed infarction		27	42.9%
Hydrocephalus		21	33.3%
3-month mRS (3–6)		30	47.6%

### Risk factors for poor outcomes at 3 months after discharge

Univariate analysis showed that several clinical factors significantly correlated with poor outcomes (Table 2). Patients with unfavorable outcomes had significantly longer hospital (p = 0.002) and NSICU (p < 0.001) stays. Poor outcomes were also associated with advanced age (p = 0.022) and higher admission systolic blood pressure (p = 0.037). Patients with lower admission GCS scores (3–5) had significantly worse outcomes (p < 0.001). Higher WFNS grades (p < 0.001) and Hunt & Hess grades (p < 0.001) were also strongly linked to poor outcomes. Among CSF parameters, elevated ApoC3 levels (p < 0.001), increased protein levels (p = 0.017), and abnormal chloride (Cl-) concentrations (p = 0.001) were significantly associated with unfavorable outcomes. Severity scores, including mFS, SEBES, and mGS, were strongly predictive of poor prognosis (all p < 0.001). Complications such as clinical vasospasm (p = 0.002), delayed infarction (p < 0.001), and hydrocephalus (p < 0.001) were significantly more frequent among patients with poor outcomes. The Shapiro-Wilk test p-values for key continuous variables are present in Supplementary Table 2.

**TABLE 2:** Univariate analysis of poor outcome at 3 months after discharge.

Variable	​	3-month mRS		
​	​	Favourable 0-2	Unfavourable 3-6	*p*-value
Hospital LOS, d		11 (9.5–13.5)	18.5 (11–33.5)	**0.002**
Sex (F)		18 (54.5)	16 (53.3)	0.923
Age, y		56.55 ± 9.60	63.17 ± 12.61	**0.022**
Systolic blood pressure, mmHg		147 (132.5–156)	160 (140.5–196.75)	**0.037**
Systolic blood pressure >190		2 (6.1)	9 (30)	**0.012**
Admission GCS	13–15	30 (90.9)	9 (30)	**<0.001**
	6–12	2 (6.1)	9 (30)	
	3–5	1 (3)	12 (40)	
WFNS grade (3-5)		8 (24.2)	21 (70)	**<0.001**
Hunt & hess grade (3-4)		5 (15.2)	23 (76.7)	**<0.001**
NSICU LOS, d		3 (1.5–5.5)	15 (7–26.5)	**<0.001**
Duration of mechanical ventilation, d		2 (1–3.5)	11 (5–28)	**<0.001**
Drinking		14 (42.4)	6 (20)	0.056
Smoking		12 (36.4)	9 (30)	0.593
Hypertension		18 (54.5)	18 (60)	0.662
Diabetes		1 (3)	3 (10)	0.538
BMI	<18.5	2 (6.1)	2 (6.7)	0.101
	<24	20 (60.6)	12 (40)	
	<28	11 (33.3)	13 (43.3)	
	≥28	0 (0)	3 (10)	
CSF examination	ApoC3, ng/mL	1,464.70 (641.40–3,201.46)	7,179.49 (2000.65–13915.86)	**<0.001**
	RBC, 10^6/L	62,100 (28,500–115000)	88,500 (32,688–101928)	0.61
	Lymphocytes, %	29.26 (15–41.5)	29.26 (12.75–34)	0.643
	Nucleated cells, 10^6/L	330 (149.5–1886.5)	590 (210.25–1,486.57)	0.825
	Glucose, mmol/L	3.59 (2.63–4.03)	4.00 (3.55–5.99)	0.059
	Protein level, mg/dL	83.3 (51.85–135.05)	135.05 (94.7–208.23)	**0.017**
	Cl^−^, mmol/L	128 (124.05–129.55)	130.2 (129.36–135.33)	**0.001**
mFS (3-4)		13 (39.4)	28 (93.3)	**<0.001**
SEBES (3-4)		18 (54.5)	28 (93.3)	**0.001**
IVH		16 (48.5)	28 (93.3)	**<0.001**
mGS		0 (0–2)	8.5 (2–19)	**<0.001**
Aneurysm Location	Internal carotid artery	3 (9.1)	2 (6.7)	0.161
	Anterior cerebral artery	20 (60.6)	11 (36.7)	
	Middle cerebral artery	5 (15.2)	6 (20)	
	Posterior circulation	5 (15.2)	11 (36.7)	
Aneurysm size	1–5 mm	15 (45.5)	9 (30)	0.113
	5.1–10 mm	17 (51.5)	16 (53.3)	
	Greater than 10 mm	1 (3)	5 (16.7)	
Coiling		11 (33.3)	10 (33.3)	1
Clipping		22 (66.7)	20 (66.7)	1
Decompression		1 (3)	10 (33.3)	**0.002**
LD		27 (81.8)	16 (53.3)	**0.015**
EVD		1 (3)	14 (46.7)	**<0.001**
Clinical vasospasm		6 (18.2)	17 (56.7)	**0.002**
Delayed infarction		5 (15.2)	22 (73.3)	**<0.001**
Hydrocephalus		3 (9.1)	18 (60)	**<0.001**

Bold values indicates the p-value that less than 0.05.

### CSF ApoC3 as an indicator of clinical severity in aSAH patients

**FIGURE 1:**
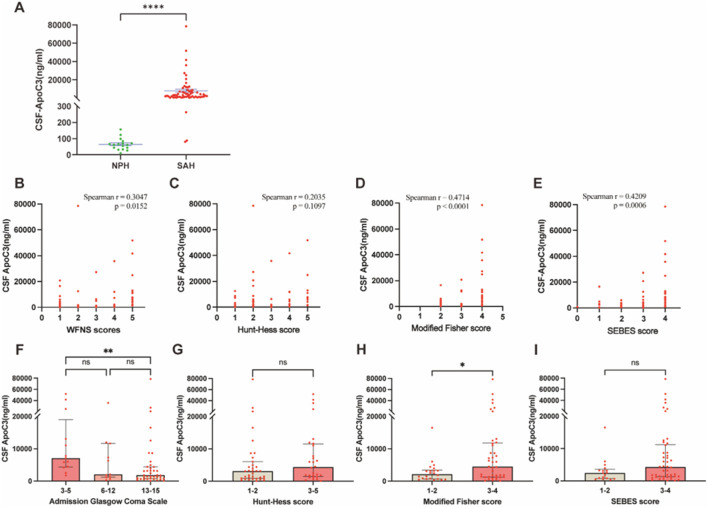
Comparison of CSF ApoC3 concentrations between NPH patients and clinical grades of aSAH. **(A)** Comparison of CSF ApoC3 levels between 63 patients with aSAH and 17 patients with NPH. **(B–E)** Spearman correlation analyses of CSF ApoC3 concentrations and clinical grades of SAH among patients with aSAH. **(F–I)** Correlation analyses between CSF ApoC3 concentrations and graded clinical scores of SAH.

CSF ApoC3 concentrations were compared between 63 aSAH patients and 17 NPH controls (Figure 1A). ApoC3 levels were significantly higher in aSAH patients compared to controls (p < 0.0001). Univariate linear regression analysis indicated significant linear correlations between ApoC3 concentrations and various clinical scores, including WFNS (p = 0.0152, Figure 1B), mFS (p < 0.0001, Figure 1D), and SEBES (p = 0.006, Figure 1E). Higher CSF ApoC3 concentrations correlated significantly with worse GCS (p < 0.01, Figure 1F) and more severe mFS groups (p < 0.05, Figure 1H). Although several clinical scores aren’t correlated with ApoC3 concentration (Figure 1C,G,I). To control for confounding factors and assess ApoC3 as an independent predictor of prognosis, multivariable logistic regression was performed, including ApoC3, age, WFNS score, Fisher score, and hydrocephalus (Table 3). The results indicated that elevated CSF ApoC3 levels were independently correlated with poor 3-month outcomes.

**TABLE 3:** Multivariate logistic regression analysis of poor outcome 3 months after discharge.

Independent variable	OR	95% CI	*p*-value
Age, y	1.05	0.91–1.22	0.488
Admission GCS	21.33	0.77–594.34	0.071
WFNS grade (3-5)	110.04	1.404–8,623.27	**0.035**
Hunt & hess grade (3-4)	198.88	2.158–17235.48	**0.022**
mFS (3-4)	1.562	0.035–69.274	0.818
SEBES (3-4)	5.826	0.133–254.59	0.36
CSF-ApoC3 (>4,463 ng/mL)	132.29	3.08–5,673.96	**0.011**
Hydrocephalus	32.07	1.25–820.85	**0.036**

Bold values indicates the p-value that less than 0.05.

### CSF ApoC3 is associated with the outcome of aSAH patients

**FIGURE 2:**
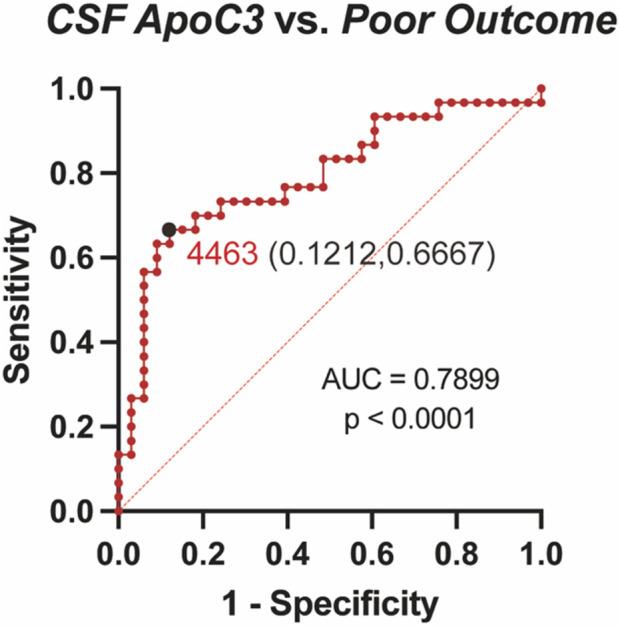
ROC curve analysis of CSF ApoC3 levels predicting poor outcomes (mRS scores 3–6) at 3 months. The results indicate that ApoC3 has strong predictive value for patient outcomes at 3 months (AUC = 0.7899, 95% CI 0.6739–0.9059). The optimal cutoff value yield a sensitivity of 0.6667 (95% CI 0.4878–0.8077) and a specificity of 0.8788 (95% CI 0.7267–0.9518).

ROC curve analysis was performed to evaluate the predictive ability of CSF ApoC3 concentrations for 3-month outcomes (Figure 2). The results demonstrated that ApoC3 levels effectively predicted poor outcomes (AUC = 0.7899, 95% CI: 0.6739–0.9059). The optimal cutoff value, determined by the Youden index, was 4,463 ng/mL, with a sensitivity of 0.6667 (95% CI: 0.4878–0.8077) and specificity of 0.8788 (95% CI: 0.7267–0.9518).

Subsequently, the optimal cutoff (4,463 ng/mL) was used to classify 63 aSAH patients into high ApoC3 and low ApoC3 groups. Univariate analysis of clinical factors between these groups is presented in Table 4.

**TABLE 4:** Univariate analysis of different ApoC3 concentration groups and aSAH clinical features.

Variable	​	CSF-ApoC3		
​	​	Low group ≤4,463 ng/mL	High group >4,463 ng/mL	*p*-value
Hospital LOS, d		12 (10–18)	17.5 (9.25–31.75)	0.31
Sex (F)		21 (53.8)	13 (54.2)	1
Age, y		59.23 ± 10.7	60.46 ± 12.96	0.685
Systolic blood pressure, mmHg		150 (133–163)	149 (139.5–192.25)	0.388
Systolic blood pressure >190		5 (12.8)	6 (25)	0.307
Admission GCS	13–15	30 (76.9)	9 (37.5)	**0.001**
	6–12	6 (15.4)	5 (20.8)	
	3–5	3 (7.7)	10 (41.7)	
WFNS grade (3-5)		12 (30.8)	17 (70.8)	**0.004**
Hunt & hess grade (3-4)		14 (35.9)	14 (58.3)	**0.118**
NSICU LOS, d		4 (2–11)	10.5 (5–23.5)	**0.018**
Duration of mechanical ventilation, d		2 (1–9)	7.5 (4–23.5)	**0.021**
Drinking		14 (35.9)	6 (25)	0.416
Smoking		14 (35.9)	7 (29.2)	0.784
Hypertension		25 (64.1)	11 (45.8)	0.194
Diabetes		3 (7.7)	1 (4.2)	0.657
BMI	<18.5	3 (7.7)	1 (4.2)	0.447
	<24	22 (56.4)	10 (41.7)	
	<28	13 (33.3)	11 (45.8)	
	≥28	1 (2.6)	2 (8.3)	
CSF examination	RBC, 10^6/L	50,000 (22,750–126000)	101,904 (58,000–101904)	0.207
	Lymphocytes, %	28 (13–39)	29.26 (11.5–37.5)	0.481
	Nucleated cells, 10^6/L	380 (130–2,364)	897.5 (210–1,486.6)	0.932
	Glucose, mmol/L	3.62 (2.59–4.03)	4.00 (3.62–5.62)	**0.016**
	Protein level, mg/dL	93.8 (48.7–157.6)	135.05 (98.78–177.28)	**0.033**
	Cl^−^, mmol/L	128 (124.4–130.7)	129.55 (129.55–135.55)	**0.009**
mFS (3-4)		20 (51.3)	21 (87.5)	**0.006**
SEBES (3-4)		25 (64.1)	21 (87.5)	0.078
IVH		24 (61.5)	20 (83.3)	0.067
mGS		2 (0–4)	7 (1.25–18.25)	**0.006**
Aneurysm Location	Internal carotid artery	3 (7.7)	2 (8.3)	0.449
	Anterior cerebral artery	22 (56.4)	9 (37.5)	
	Middle cerebral artery	5 (12.8)	6 (25)	
	Posterior circulation	9 (23.1)	7 (29.2)	
Aneurysm size	1–5 mm	16 (41)	8 (33.3)	0.802
	5.1–10 mm	19 (48.7)	14 (58.3)	
	Greater than 10 mm	4 (10.3)	2 (8.3)	
Coiling		12 (30.8)	9 (37.5)	0.784
Clipping		27 (69.2)	15 (62.5)	0.784
Decompression		3 (7.7)	8 (33.3)	**0.015**
LD		29 (74.4)	14 (58.3)	0.265
EVD		9 (23.1)	6 (25)	1
Clinical vasospasm		12 (30.8)	11 (45.8)	0.285
Delayed infarction		10 (25.6)	17 (70.8)	**0.001**
Hydrocephalus		8 (20.5)	13 (54.2)	**0.012**
3-month mRS (3–6)		10 (25.6)	20 (83.3)	**<0.001**

Bold values indicates the p-value that less than 0.05.

Patients in the high ApoC3 group exhibited significantly worse clinical scores upon admission. Specifically, they showed lower GCS scores (p = 0.001), higher WFNS grades (p = 0.004), higher mFS scores (p = 0.006), and higher mGS scores (p = 0.006). Moreover, patients with elevated ApoC3 experienced significantly longer NSICU stays (p = 0.018) and longer durations of mechanical ventilation (p = 0.021). Regarding surgical interventions, no significant differences existed between the two groups in the rates of coiling, clipping, or LD/EVD drainage. However, patients undergoing decompression were significantly more likely to have elevated ApoC3 (p = 0.015). CSF analysis revealed significantly increased glucose, protein, and Cl^−^ levels in the high ApoC3 group. Notably, the high ApoC3 group exhibited significantly worse 3-month functional outcomes (p < 0.001). Additionally, complications such as delayed infarction (p = 0.001) and hydrocephalus (p = 0.012) occurred significantly more often in the high ApoC3 group.

### aSAH patients with elevated ApoC3 experienced worse outcomes at discharge and three months

**FIGURE 3:**
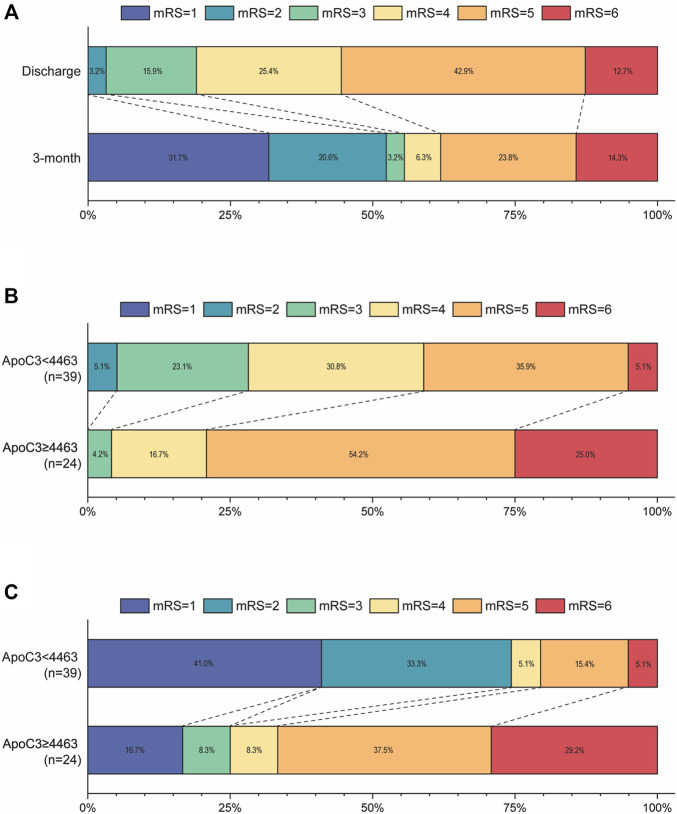
Stacked bar charts illustrating outcomes of aSAH patients based on mRS scores at discharge and at 3 months. **(A)** Distribution of mRS scores among 63 aSAH patients at discharge and at 3 months. **(B)** Percentage distribution of mRS scores at discharge among aSAH patients stratified by ApoC3 levels. **(C)** Percentage distribution of mRS scores at 3 months among aSAH patients stratified by ApoC3 levels.

Three months post-discharge, the proportion of patients with poor outcomes (mRS 3–6) decreased from 96.9% at discharge to 47.6% at 3 months (Figure 3A). However, patients in the high ApoC3 group (CSF ApoC3 >4,463 ng/mL) had consistently poor mRS scores at discharge, with 100% scoring 3–6 (Figure 3B). Even after 3 months of rehabilitation, poor outcomes remained prevalent (83.3%) in the high ApoC3 group, while decreasing to 25.6% in the low ApoC3 group (Figure 3C).

## Discussion

In this study, experimental and clinical data demonstrated that among CSF parameters, elevated ApoC3 levels were significantly associated with adverse prognosis and an increased risk of complications. Using an optimal cutoff value of 4,463 ng/mL, patients in the high ApoC3 group exhibited significantly worse functional outcomes at 3 months (p < 0.001) and experienced more complications, particularly delayed infarction (p = 0.001) and hydrocephalus (p = 0.012). Therefore, promptly monitoring CSF ApoC3 levels and taking appropriate preventive and therapeutic measures may enhance outcomes for patients with aSAH.

It has long been recognized that lipid metabolism is associated with the formation and rupture of intracranial aneurysms, contributing to aSAH [10–13]. Several studies have indicated that blood lipid levels are significant predictors of prognosis in patients with aSAH15. ApoC3, a glycoprotein primarily synthesized in the liver, is a crucial regulator of TRL metabolism [11]. ApoC3 is a glycoprotein primarily synthesized in the liver and serves as a key regulator of TRL metabolism. Previous studies have demonstrated that ApoC3 is significantly involved in the pathogenesis, progression, and prognosis of vascular diseases. For instance, loss-of-function mutations in ApoC3 can decrease the risk of atherosclerotic cardiovascular disease by approximately 40% and protect against coronary heart disease, consequently increasing longevity [14]. However, the relationship between ApoC3 and aSAH remains unclear.

In our study, we found that CSF ApoC3 levels in aSAH patients were significantly negatively correlated with clinical scores, including the WFNS score (p = 0.0152), mFS (p < 0.0001), and SEBES score (p = 0.006), indicating that elevated CSF ApoC3 may reflect more severe clinical symptoms in patients with aSAH. Moreover, univariate analysis of various clinical factors confirmed that CSF ApoC3 levels above 4,463 ng/mL effectively predicted poor 3-month outcomes after discharge, yielding an area under the curve (AUC) of 0.7899. Our findings are consistent with previous studies indicating that elevated blood lipid levels predict adverse outcomes in aSAH patients [11].

### Future directions

The negative correlation observed between elevated CSF ApoC3 levels and poor prognosis may be related to inflammation, oxidative stress, and dysregulated lipid ratios. Previous studies have demonstrated that ApoC3 mediates NLRP3 inflammasome activation in human monocytes and induces reactive oxygen species production via caspase-8 activation and Toll-like receptor (TLR) 2 and 4 dimerization, processes that impede endothelial regeneration and promote kidney injury [15], and previous study has shown that elevated inflammatory level in the CSF are associated with the development of complication following SAH [16]. Thus, elevated CSF ApoC3 may exacerbate inflammatory responses and oxidative stress, thereby aggravating brain injury in aSAH patients. Additionally, ApoC3 accumulates in circulating blood and performs an essential function in TRL metabolism. Elevated CSF ApoC3 may suggest increased penetration of peripheral substances into the brain, contributing to more severe pathology. Changes in lipid ratios associated with elevated CSF ApoC3 levels may also contribute to secondary injury following SAH [10].

Moreover, it is recognized that lipid metabolites in CSF are associated with the risk, complications, and outcomes in patients with aSAH. Levels of lipid peroxides are positively correlated with an increased incidence of symptomatic vasospasm [17]. Total cholesterol levels negatively correlate with the risk of delayed infarction and mortality in aSAH patients [18]. Elevated CSF ApoC3 may increase the prevalence of complications (particularly delayed infarction and hydrocephalus) in aSAH patients by altering lipid metabolism, leading to vasospasm, neuroinflammation, blood-brain barrier (BBB) disruption, and endothelial fragility [18–21]. Collectively, these mechanisms may explain how elevated CSF ApoC3 worsens clinical outcomes in aSAH patients.

Increased ApoC3 may originate from circulating blood, with ApoC3 levels rising in parallel with total protein or albumin quotients, reflecting the extent of BBB disruption. Regardless of whether ApoC3 is synthesized in the brain or derived from peripheral circulation, the clear correlation between ApoC3 levels and the prognosis of aSAH patients supports its utility as a valuable marker for clinical decision-making. In addition, considering that hydrocephalus and cerebral edema are crucial indicators for evaluating aSAH patients [16], we selected NPH patients with hydrocephalus and normal CSF composition as the control group. It should be noted that NPH patients might exhibit abnormal CSF dynamics. However, due to ethical constraints, obtaining CSF from healthy individuals is not feasible; thus, CSF form NPH patients is currently the most suitable control in clinical practice without increasing patient burden. And CSF sample from NPH patients is chosen as the standard control group in aSAH research [22].

## Conclusion

This study indicates that ApoC3 may serve as a potential biomarker for predicting DCI and hydrocephalus. With an AUC of 0.7899 and an optimal cutoff value of 4,463 ng/mL, measuring CSF ApoC3 levels during the acute stage of aSAH could help clinicians determine the timing and type of preventive and therapeutic interventions, thereby potentially reducing complication rates and improving patient outcomes. However, given the limited sample size and coarse sampling time, further validation through larger sample sizes, continuous monitoring ApoC3 level in CSF, and multicenter studies is necessary before clinical application.

## Data Availability

The datasets for this article are not publicly available due to concerns regarding patient anonymity. Requests to access the datasets should be directed to the corresponding author.
