# Supplementary material for: Elevated ApoC3 levels in cerebrospinal fluid predict poor outcomes in patients with aneurysmal subarachnoid hemorrhage
Source: Exp Biol Med (Maywood). 2026 Apr 17;251:10827. doi: 10.3389/ebm.2026.10827 (PMC13132793; doi:10.3389/ebm.2026.10827)
Supplement: Supplementary file 1 [file Table1.docx]

Supplementary Table 1. Demographic and Clinical Data of 17 patients with NPH

|  | | **Average/median/N** | **SD/IQR/%** |
| --- | --- | --- | --- |
| **Sex (F)** | | **8** | **47%** |
| **Age, y** | | 68 | ±15 |
| **Systolic blood pressure, mmHg** | | 130 | (99-176) |
| **Admission GCS** | 13-15 | 13 | 76% |
|  | 6-12 | 3 | 18% |
|  | 3-5 | 1 | 6% |
| **Drinking** | | 4 | 24% |
| **Smoking** | | 5 | 29% |
| **Hypertension** | | 7 | 41% |
| **Diabetes** | | 5 | 29% |
| **BMI** | | 22.89 | ±2.18 |
| **CSF pressure,** mmH2O | | 118 | 90-160 |
| **CSF Examination** | RBC, 10^6/L | 114 | 0-2000 |
|  | Nucleated cells, 10^6/L | 2 | 0-8 |
|  | Glucose, mmol/L | 3.86 | 3.04-4.25 |
|  |  |  |  |

|  |  | **Average/median/N** | **SD/IQR/%** |
| --- | --- | --- | --- |
| **CSF Examination** | Protein level, mg/dL | 36.3 | 28-42.6 |
|  | Cl^-^, mmol/L | 125.5 | 121-127.8 |
