# Supplementary material for: Elevated ApoC3 levels in cerebrospinal fluid predict poor outcomes in patients with aneurysmal subarachnoid hemorrhage
Source: Exp Biol Med (Maywood). 2026 Apr 17;251:10827. doi: 10.3389/ebm.2026.10827 (PMC13132793; doi:10.3389/ebm.2026.10827)
Supplement: Supplementary file 2 [file Table2.docx]

Supplementary Table 2. Results of Shapiro–Wilk normality test

| **Independent Variable** | | ***p*-value of Shapiro-Wilk test** | **Data presentation** |
| --- | --- | --- | --- |
| **Hospital LOS, d** | | <0.001 | IQR |
| **Age, y** | | 0.141 | SD |
| **Systolic blood pressure, mmHg** | | 0.002 | IQR |
| **Duration of mechanical ventilation, d** | | <0.001 | IQR |
| **CSF Examination** | RBC, 10^6/L | <0.001 | IQR |
|  | Lymphocytes, % | <0.001 | IQR |
|  | Nucleated cells, 10^6/L | <0.001 | IQR |
|  | Glucose, mmol/L | <0.001 | IQR |
|  | Protein level, mg/dL | <0.001 | IQR |
|  | Cl^-^, mmol/L | <0.001 | IQR |
|  | ApoC3, ng/ml | <0.001 | IQR |
